# From knowledge generation to knowledge verification: examining the biomedical generative capabilities of ChatGPT

**DOI:** 10.1016/j.isci.2025.112492

**Published:** 2025-04-21

**Authors:** Ahmed Abdeen Hamed, Alessandro Crimi, Magdalena M. Misiak, Byung Suk Lee

**Affiliations:** 1 MGEN – College of Engineering, Northeastern University Miami, Miami, FL 33127, USA; 2 The Institute for Experiential AI, Northeastern University, Boston, MA 02115, USA; 3 CASCI Laboratory, Binghamton University, Binghamton, NY 13902, USA; 4 AGH University of Krakow, Faculty of Informatics, 30-059 Krakow, Poland; 5 Department of Physiology and Biophysics, Howard University, Washington, DC 20059, USA; 6 Department of Computer Science, University of Vermont, Burlington, VT 05405, USA

**Keywords:** Health sciences, Medicine, Health informatics, Health technology

## Abstract

The generative capabilities of LLM models offer opportunities for accelerating tasks but raise concerns about the authenticity of the knowledge they produce. We present a computational approach that evaluates the factual accuracy of biomedical knowledge generated by an LLM. Our approach consists of generating disease-centric associations and verifying them using biomedical ontologies. Using ChatGPT, we designed prompt-engineering processes to establish linkages between diseases and related drugs, symptoms, and genes, and assessed consistency across multiple ChatGPT models (e.g., GPT-4, GPT-4o, and GPT-4o-mini). Results demonstrate high accuracy in identifying disease terms (88%–97%), drug names (90%–91%), and genetic information (88%–98%). Symptom term identification was lower (49%–61%) due to informal symptom descriptions. Verification reveals coverage of 89%–91% for disease-drug and disease-gene pairs; symptom-related associations show lower coverage (49%–62%). Despite high term accuracy, generated IDs were often invalid or redundant. GenAI tools can be reliable if used with care. Retrieval Augmented Generation (RAG) may enhance reliability.

## Highlights

• ChatGPT can generate disease-centric biomedical associations using LLM prompts
• Ontologies help verify terms for diseases, drugs, symptoms, and genetic associations
• PubMed is used to check the validity of associations generated by LLMs
• ChatGPT shows variable consistency in regenerating biomedical associations across its own models

## Graphical abstract

**Figure:**
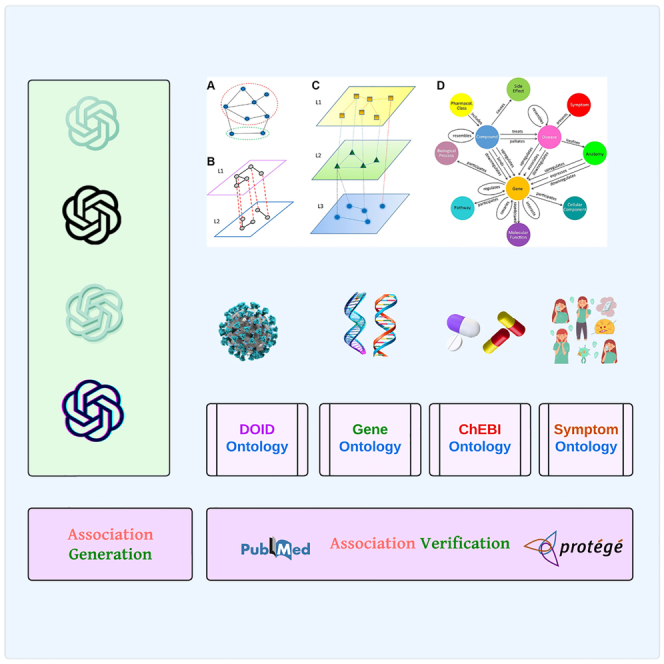

## Introduction

Large language models (LLMs) possess impressive generative capabilities, presenting opportunities to accelerate various tasks while also raising concerns about the reliability of the knowledge they generate. Concerned with the growing threat to scientific authenticity since the emergence of ChatGPT,^1^ our previous perspective laid out a research agenda focused on safeguarding authenticity.^2^ This agenda addressed two key issues: combating fake science and verifying the factuality of AI-generated content. The first issue, fighting fake science, has become increasingly urgent as ChatGPT’s generative capabilities can produce convincing but fabricated scientific articles. In our earlier work, we introduced the xFakeSci algorithm, a machine learning approach that distinguished fake/AI-generated articles from real publications with high precision.^3^ This development underscores the need for robust mechanisms to detect and filter out misleading content. The second issue, which is the focus of this paper, is the verification of the factuality of content generated by ChatGPT. Here, we extend our previous efforts by developing algorithmic approaches to assess and verify biomedical associations. Our experiments leverage relevant biomedical ontologies and PubMed abstracts to systematically evaluate the factual accuracy of the generated content.

In an era marked by the rapid adoption of generative AI tools, fact-checking and knowledge verification are essential safeguards against hallucination and misinformation. This review traces the evolution of fact-checking, from its pre-ChatGPT foundations to its current role in addressing challenges posed by advanced generative models, especially in biomedical applications.

Prior to the emergence of ChatGPT, research efforts focused on examining misinformation and developing fact-checking strategies. Several studies investigated the authenticity of information and explored fact-checking methods to mitigate misinformation risks.^4^^,^^5^^,^^6^^,^^7^ Surveys provided a comprehensive overview of automated fact-checking models and databases,^8^^,^^9^ while others used natural language processing techniques to verify news articles and social media content.^10^^,^^11^ Additionally, machine learning approaches were applied to combat fake news, fake science, and fake social media posts.^12^^,^^13^^,^^14^^,^^15^ The urgency of these efforts was further underscored during the global pandemic, as misinformation raised significant health and public safety concerns.^5^^,^^16^^,^^17^^,^^18^

The release of ChatGPT, alongside other generative AI and large language models, expanded research opportunities while intensifying concerns about misinformation. On one hand, these models have unlocked new scientific possibilities^19^^,^^20^^,^^21^^,^^22^; on the other, they have raised issues regarding hallucinations and the lack of citations, which challenge scientific authenticity.^23^^,^^24^^,^^25^^,^^26^ In response, new fact-checking approaches emerged. For instance, one study addressed the verification of simulated medical abstracts by examining disease and gene names,^27^ while another explored fact-checking solutions to mitigate risks associated with factuality in large language models.^28^ Additional systems, such as LLM-Augmenter, have been designed to cross-verify content against external resources, and deep-learning classifiers have been used to check AI-generated radiology reports.^29^^,^^30^ Moreover, SelfCheckGPT has been developed to assess factuality on a sentence-by-sentence basis by ranking text chunks.^31^

Biomedical research has benefited from early models such as BioBART, which assisted with Named Entity Recognition (NER), Entity Linking, and Question Answering tasks at a limited scale.^32^ Following ChatGPT’s debut, studies began exploring its utility in biomedical question-answering.^33^^,^^34^ Concurrently, researchers have investigated the use of large language models to generate knowledge directly or via retrieval-augmented generation (RAG) methods. RAG integrates contextual prompts to enhance the freshness and accuracy of the generated information.^35^^,^^36^^,^^37^^,^^38^ For example, one study employed ChatGPT as a decision support system for self-screening by embedding screening guidelines into hypothetical cases.^39^ Another used RAG-based prompt engineering to extract structured representations of drug combinations from clinical trials.^40^ Similar approaches have also improved PubMed’s retrieval capabilities.^41^

Beyond biomedical applications, large language models have been evaluated for their fact-checking abilities in news and multilingual settings. Comparative studies of ChatGPT, Bing AI CoPilot, and Gemini (formerly Bard) have highlighted both their potential and the continuing need for human oversight in news verification.^1^^,^^42^^,^^43^^,^^44^ In the multilingual arena, research employing techniques such as zero-shot, chain-of-thought, and cross-lingual prompting has shown that languages with fewer resources may sometimes yield more accurate fact-checking results.^45^ Additionally, studies have underscored the importance of developing guidelines for using AI in fact-checking news headlines.^46^

The goal of our work is to test the generative capabilities of ChatGPT to generate disease-centric biomedical terms and associations and perform various verification processes to assess the factuality of such associations. There are three objectives under this goal.

**Objective 1:** To perform semantic term verification using relevant biomedical ontologies for disease, drugs, symptoms, and genes.

**Objective 2:** To perform the automated verification of association using against the biomedical literature, specifically, PubMed abstracts.

**Objective 3:** Assess ChatGPT's consistency in generating knowledge using independent processes and various ChatGPT models: gpt-turbo, gpt-4o, gpt-4, and gpt-4o-mini.

## Results

We present the results of the experiments in evaluating ChatGPT’s capabilities in the following key tasks.

(1) Verifying the correctness of the biomedical terms that make up the associations (i.e., disease, symptom, drug, and genes);
(2) Verifying the associations' linkage against biomedical literature from different periods;
(3) Testing the randomness of ChatGPT by generating simulated articles using various ChatGPT models.

### Task 1 – Verification of the correctness of biomedical terms

We evaluated the names of the three types of associations generated: disease-drug, disease-symptom, and disease-gene/genetic process that made up the ChatGPT-generated associations using domain-specific ontologies as ground truth. The verification of the terms that make up the generated associations was checked against the DOID ontology for disease terms, the ChEBI ontology for the drug terms, the SYMPTOM ontology for the symptom terms, and the GO ontology for the genetic terms (gene names and genetic processes). The encoding of those ontologies offers means of literal and semantic matching, which offers fair means of comparisons. For instance, the (“hypertension”) disease term in the DOID ontology (“DOID:10763”).^47^ Additionally, the ontology entity of this term also includes the list of synonyms (“HTN [EXACT], hyperpiesia [EXACT], hypertensive disease [RELATED], vascular hypertensive disorder [EXACT]”), which are also checked during the algorithmic process. Hence, the claim of a semantic verification process.

#### Tasks 1.1 – Verification of disease terms

The task of generating disease terms was common across three types of associations. The verification result of the terms in the three types of associations are as follows.

(1) For disease-drug associations, the literal matching process verified 93% of disease terms, while the semantic matching verified 87% of the generated names. Combined, 98% of disease names were successfully verified.
(2) For disease-symptom associations, the literal matching verified 97% of the disease terms, while the semantic matching verified 82% of the generated terms. Combined, 99% of disease names were successfully verified.
(3) For disease-gene associations, the literal matching verified 88% of the disease terms, while semantic matching verified 97% of the generated terms. Due to the high percentage of verification, the task of a combined matching was omitted.

#### Tasks 1.2 – Verification of non-disease terms

Here, we summarize the results of ChatGPT generating correct drugs, symptoms, genes, and genetic processes as part of the associations.

(1) **Drug names:** the literal matching verified 90% of drug names, with 90% verified through synonym matching. The combined verification rate was 91%.
(2) **Symptom names:** literal matching verified 49% of symptom names, with an additional 25% verified through semantic matching. The combined verification rate was 61%.
(3) **Genetic processes and gene names:** the verification resulted in the verification of 80% of the gene names and 97% of the genetic processes.

**Figure 1:**
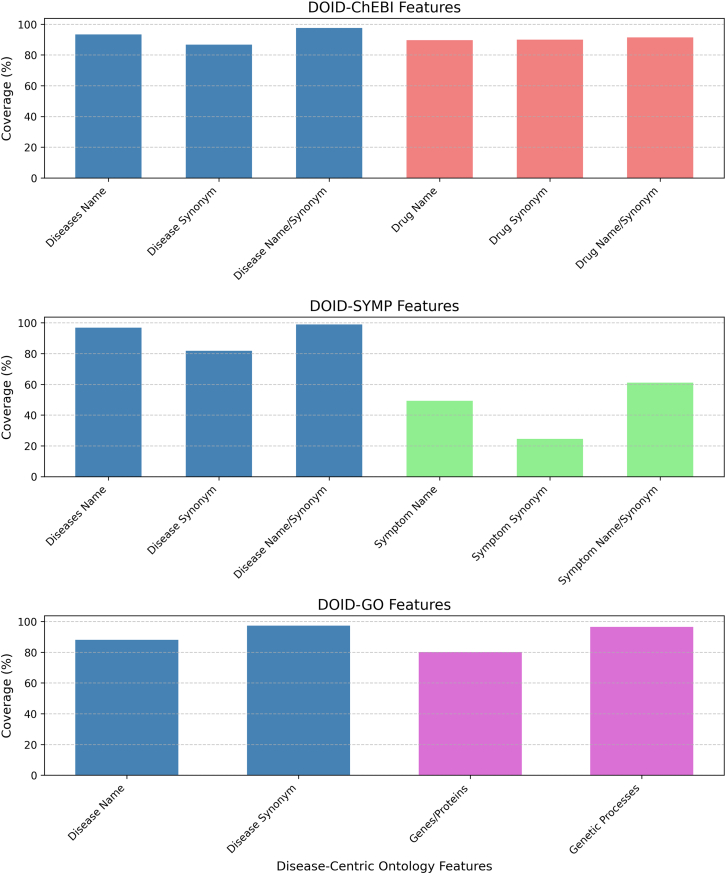
Accuracy of DOID-ChEBI, DOID-SYMP, and DOID-GO associations across various features

These findings demonstrate the strong capability of ChatGPT in generating biomedical terms that align closely with biomedical ontology as one of the most authentic sources of ground truth. Even in the case of the symptom terms, where performance was notably lower, this does pose a significant concern from the point of view of our study. Table 1 captures the statistics that summarize this task, and the results are shown in Figure 1. For Task 2, the results to be discussed forward will show that, although not identified in the specialized ontology, there was a noticeable improvement when searching the literature for association links, which also included symptom terms.

**Table 1:** Verification of entity names and types of ChatGPT-generated associations of disease-symptoms-drug-gene using biomedical ontologies (DOID for diseases, SYMP for disease symptom names, ChEBI for drug names, and GO for genetic processes and gene names)

Category	Feature Verified	Accuracy (%)
DOID-ChEBI associations	Diseases name	93.37
	Disease synonym	86.70
	Disease name/synonym	97.60
	Drug name	89.52
	Drug synonym	89.98
	Drug name/synonym	91.43
DOID-SYMP associations	Diseases name	96.83
	Disease synonym	81.87
	Disease name/synonym	98.87
	Symptom name	49.29
	Symptom synonym	24.50
	Symptom name/synonym	61.14
DOID-GO associations	Disease name	88.12
	Disease synonym	97.36
	Genes/proteins	80.21
	Genetic processes	96.47

The disease-centric links generated were DOID–ChEBI, DOID–SYMP, and DOID-GO.

### Task 2: Verification of the reliability of biomedical association links

While domain-specific ontologies offer the verification of individual entities of the associations, they do not offer the means of verifying the actual relationships between the individual terms. To address this limitation, we utilized the biomedical literature as another reputable source of ground truth to verify the various associations. Thus, we constructed three datasets, each to verify one of the three types of associations, that is, disease-drug, disease-genetic information, and disease-symptom. Because the GO ontology does not reference the direction of the gene terms in the ontology, in another resource known as the GO ontology annotation,^48^^,^^49^^,^^50^ we prompted ChatGPT to produce associations that link a disease to two pieces of genetic knowledge—the GO term, which is the genetic process itself, and the gene name, which is the product of the actual process. This requires the validation of mapping the biological process to its product, which is also included in the results.

In addition, to examine the effect of publication longevity, each dataset was divided into three 5-year periods spanning 2009 to 2024. The verification processes consider the literature coverage as a means of being viable. The premise is that if an association occurs frequently in a set of PubMed abstracts, then this makes it “verified.” The research stops at this step and does not further explore the semantics of such associations. As mentioned earlier, if an association was not covered in the literature, then this means it is yet to be verified. In other words, it does not label an association as correct or incorrect. The experiments revealed a consistent trend of improved verification coverage across all types of association over the three periods. Specifically.

(1) **Disease-drug association** is verified with coverage rates of 86%, 88%, and 90%, respectively, over the three periods.
(2) **Disease-gene association** is verified with coverage rates of 83%, 83%, and 89%, respectively, over the three periods.
(3) **Disease-symptom association** is verified with coverage rates of 49%, 53%, and 62%, respectively, over the three periods.
(4) **Genetic process-gene association** is verified with the coverage improved from 23% in the first period to 83% and 89% in the second and third periods, respectively, over the three periods.

Additionally, we further analyzed the frequency of publications supporting each type of association to measure the level of support from the literature over time. The results summarized in Table 2 indicate an increasing trend of publication support over the three periods of time (2009–2014, 2015–2019, and 2020–2024).

(1) **Disease-gene association** is supported by an average of 90, 82, and 214 publications, respectively, over the three periods.
(2) **Disease-drug association** is supported by an average of 31, 56, and 107 publications, respectively, over the three periods.
(3) **Disease-symptom association** exhibits the lowest support, with an average of 9, 14, and 31 publications, respectively, over the three periods. However, it still follows an upward trend.

**Table 2:** Average co-occurrences across time periods for disease associations

Association Type	Time Period	Average Frequency
Disease – gene	2009–2014	90.48
	2015–2019	82.54
	2020–2024	214.38
Disease – drug	2009–2014	30.71
	2015–2019	56.37
	2020–2024	107.34
Disease – symptom	2009–2014	9.17
	2015–2019	13.96
	2020–2024	31.40

**Figure 2:**
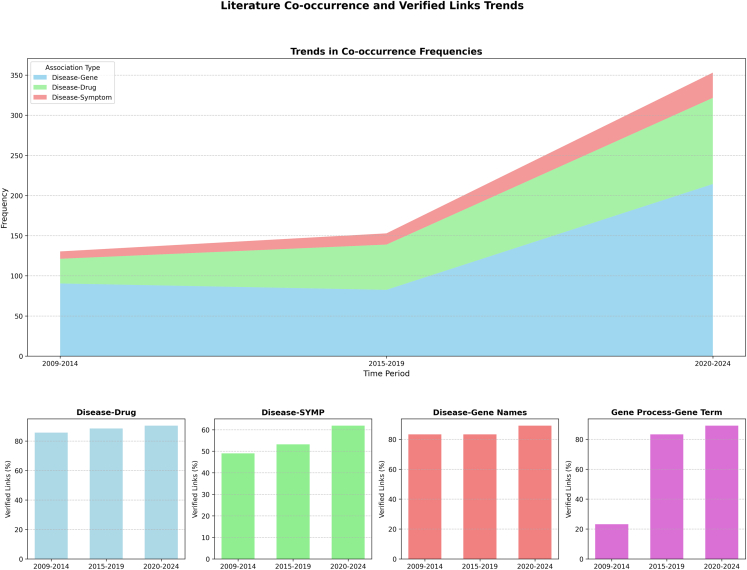
Combined visualization of average co-occurrences (line chart) and literature co-occurrence statistics (bar charts) The top plot shows trends in co-occurrences over time, while the bottom plot compares verified and unverified links for different association types.

The results from the two tasks demonstrate the high accuracy of the biomedical terms that make up the ChatGPT-generated associations, and also show an increasing coverage trend over time as captured in Table 3. The trend suggests that recent publications contribute more robustly to the verification of biomedical associations, as shown in Figure 2. On the one hand, the ChatGPT associations depict an evolutionary picture of the knowledge accumulated in biomedical literature over time. On the other hand, the verification process may also reflect on the type of knowledge ChatGPT may contribute and an indication of how the pre-training processes of ChatGPT have taken place.

**Table 3:** Literature co-occurrence statistics for disease-drug, disease-symptom, disease-gene name, and gene process associations

Association Type	Time Period	Unverified Links (%)	Verified Links (%)
Disease – drug	2009–2014	14.29	85.71
	2015–2019	11.53	88.47
	2020–2024	9.52	90.48
Disease – symptom	2009–2014	51.02	48.98
	2015–2019	46.83	53.17
	2020–2024	38.08	61.92
Disease – gene	2009–2014	16.74	83.26
	2015–2019	16.74	83.26
	2020–2024	10.85	89.15
Gene process – gene term	2009–2014	76.84	23.16
	2015–2019	16.74	83.26
	2020–2024	10.85	89.15

### Task 3: Verification of the association consistency against ChatGPT-simulated abstracts by various models

**Figure 3:**
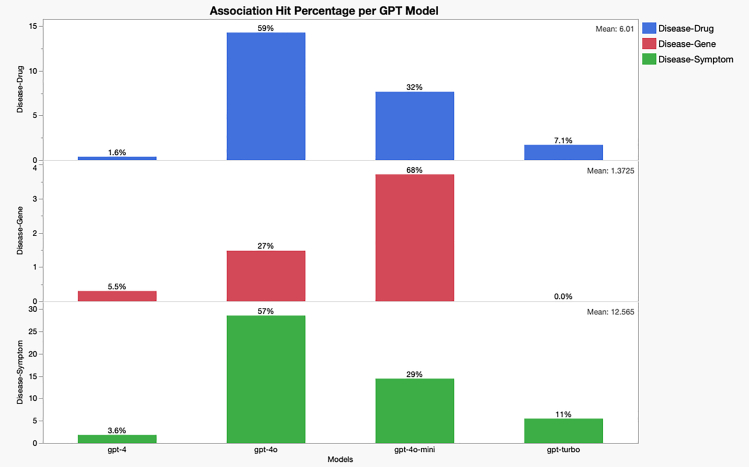
A model performance comparison, as a means of self-consistency, for the associations generated from independent prompts

To assess how consistent (or random) the ChatGPT-generated associations are, we generated disease-centric simulated abstracts using various ChatGPT models. Specifically, we prompted four ChatGPT models — ChatGPT-4, ChatGPT-4turbo, ChatGPT-4o, and ChatGPT-4omini—to generate simulated abstracts centered on human diseases. Due to the computational cost of this task, we limited the generation to approximately 5,000 abstracts per model. Figure 3 shows the number of hits per model for three types of associations in three layers: the top layer in blue is for disease-drug, the middle layer in red shows the drug-genes, while the bottom layer in green is for the disease-symptom. The verification process is summarized in Table 4.

(1) **Disease-drug association** achieved coverage rate of (1%–15%);
(2) **Disease-gene association** achieved coverage rate of (1%–4%);
(3) **Disease-symptom association** achieved coverage rate of (2%–29%).

**Table 4:** Combined statistics of disease-drug, disease-gene, and disease-symptom associations checked against the ChatGPT-generated simulated biomedical abstracts

Model	Count	Percentage (%)	Association type
chatgpt_4_model_count	10	0.38	Disease-Drug
model_turbo_chatgpt_count	45	1.71	Disease-Drug
chatgpt-4o-mini-model	201	7.66	Disease-Drug
chatgpt-4o-model	375	14.29	Disease-Drug
chatgpt_4_model_count	15	0.30	Disease-Gene
model_turbo_chatgpt_count	0	0.00	Disease-Gene
chatgpt-4o-mini-model	186	3.71	Disease-Gene
chatgpt-4o-model	74	1.48	Disease-Gene
chatgpt_4_model_count	100	1.83	Disease-Symptom
model_turbo_chatgpt_count	299	5.47	Disease-Symptom
chatgpt-4o-mini-model	788	14.42	Disease-Symptom
chatgpt-4o-model	1560	28.54	Disease-Symptom

**Figure 4:**
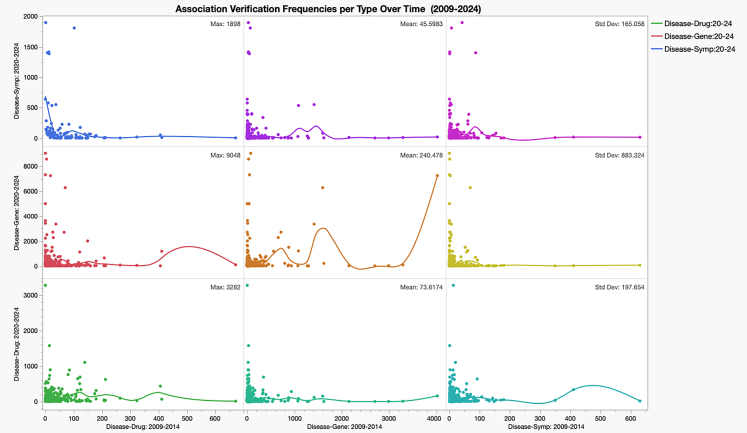
Time and summary statistics of the association coverage for three types(disease-symptom), (disease-drug), and (disease-gene)

While these coverage rates appear modest compared to benchmarks against much larger biomedical literature datasets (spanning 250,000 to 650,000 abstracts), the disparity is likely attributable to the smaller dataset size in this evaluation. Notably, the disease-symptom associations exhibited the highest match rates, a result that contrasts with their lower performance in comparisons against biomedical literature datasets. These findings underscore the potential of ChatGPT models to identify consistent disease-symptom links, though further investigation is required to validate the correctness of these associations in more focused contexts. Figure 4 shows the evaluation of associations across the various models and summary statistics for each type over three 5-year publication periods.

## Discussion

In this work, we tested the capabilities of ChatGPT to generate biomedical associations as building blocks for more complex data models such as biomedical networks and knowledge graphs. Specifically, we designed a prompt-engineering algorithm that produces human disease-centric associations in the context of symptoms, drugs, and genetics. The algorithm is prompted to generate association terms that match the corresponding specialized ontology, namely, DOID, ChEBI, SYMPTOM, and GO ontology. The prompt also provided a shot as an example of what is to be produced for a valid association. Each association was to be between two terms, a source and a target, where each term is encoded by a term ID and a name (or an instance of its synonyms). Note that the research was driven by the verification of the terms that make up the generated associations using the mentioned ontologies. The associations were verified against the biomedical literature using instances of PubMed abstracts from different periods. Here, we discuss our observations along with some anecdotal evidence in each of the verification tasks performed.

### Term correctness verification

The most striking observation is in the outcome of low coverage or symptom term verifications using the SYMP ontology. Our manual analysis revealed several critical reasons for the challenges encountered when matching symptom terms from the ontology during the verification process: (1) the language discrepancy posed significant challenges. ChatGPT predominantly generates symptoms described in social or layman’s terms that resonate more with general audiences rather than the specialist terminology employed in biomedical ontologies. Unlike drug terminology, which typically has multiple recognized names (generic, brand, and so forth), symptoms frequently lack such standardized variations. Examples illustrating this challenge include terms such as “itchy blisters” versus “blister,” "seizures" instead of "febrile convulsion," "facial redness" instead of "inflammation," and "sad mood" versus “depression.” (2) ChatGPT tended to generate overly verbose or too detailed symptom descriptions, complicating direct matching with succinct ontology terms or their synonyms. Examples include phrases such as "rapid weight loss" versus simply "weight loss," "swelling in joints," and "swollen lymph nodes," where simpler ontology terms exist. (3) instances occurred where multiple symptoms were combined into one description, preventing direct term-to-term matching. A notable example was combining “bulls-eye/bull’s eye” (typically classified as a lesion) with "rash," thus complicating the ontology-based verification. (4) Inconsistencies in punctuation and a lack of synonym availability within the ontology further hindered symptom identification, underscoring the importance of advanced semantic matching mechanisms. These issues also contributed to the verification of disease-symptom associations.

### Association reliability and consistency verification

Association verification against the biomedical literature results in a positive outcome of very high coverage for some associations. This observation offers some confidence knowing that ChatGPT has certain knowledge that may be considered the fundamentals of science, common knowledge, or frequently studied associations; examples of such associations are the disease-drug association between diabetes mellitus and insulin, which was covered by 3000+ co-occurrences; the disease-gene association between breast cancer and ataxia telangiectasia mutated (ATM), where 9000 co-occurrences pointed to it that the ATM gene may cause the breast cancer. Generating such associations may summarize the basic building blocks in human diseases, which is the ultimate objective of this study. It may also trigger the incremental generation of associations while performing verification, to construct a more comprehensive human disease landscape.

### Association consistency verification

When performing the association consistency verification, we observed that some of the frequencies are only a single occurrence in a publication abstract. These low-coverage associations may be coincidental in most cases. However, after investigating various instances, we found, for example, that the association between non-small cell lung cancer and lapatinib as a disease-drug type is proven correct. Further, we also found that the same drug was approved by the FDA to be repurposed for breast cancer in combination with a chemotherapy drug known as capecitabine,^51^ which presents a piece of drug repurposing evidence.

Other similar observations, the association between hypertensive heart disease and losartan also had a very weak coverage in the literature. Losartan, as a standalone drug according to Xu et al.^52^ or in combination with hydrochlorothiazide according to Suzuki et al.,^53^ plays a significant role in treating cardiovascular diseases, including hypertension and heart diseases. Both publications became available in the period from 2009 to 2015. We hypothesize that ChatGPT may have generated this knowledge from another source of evidence that is thought to be significant. It is important to study the associations of weak coverage and further explore such evidence to understand its value.

When we tested if the publication date was a factor in the training of ChatGPT, we found that indeed the publication date played a role in the verification of the knowledge generated by ChatGPT. Specifically, we found more coverage for associations from publications published in recent years (2020–2024) than earlier. The coverage was significantly lower if the publication was old by a decade or more. However, the ChatGPT generated associations were only found in older literature than recent, as in Xu et al., and Suzuki et al.^52^^,^^53^

The way we used different ChatGPT models to generate simulated abstracts and to measure the coverage also proved that ChatGPT’s ability to generate associations was based on a certain ground, and it was not random. Although the overlap of associations in the generated abstract was low, it was a good test of trust. It is important to acknowledge that the abstracts were generated in the most generic way based on their pre-trained knowledge about human diseases, symptoms, genetics, and drugs. More importantly, the abstracts were also generated in an entirely independent process and produced a different type of output (i.e., structured associations).

### Methods

#### Data generation via prompt engineering

To test the knowledge generation capabilities of ChatGPT, we used means of prompt engineering via the APIs. The purpose was to instruct ChatGPT to generate various types of disease-centric term associations to enable the verification process. These term associations are the basic building blocks of more complex forms of knowledge represented in the knowledge networks. Generating and verifying various types of associations makes the task of knowledge verification easy and efficient by decomposing the verification tasks to fine-grained unit of term associations, thereby reducing the effort to build a large and complex knowledge graph. Specifically, we instructed ChatGPT to generate 5000 associations between disease on one side, and gene, symptom, and drug, respectively, on the other side. Recall that the main idea for verification is to verify (1) whether the terms of the associations are verifiable from the corresponding ontology and (2) whether the actual association instances are rooted in the literature. To this end, the prompt included generating pairs of verifiable ontology-terms with their IDs.

**Figure 5:**
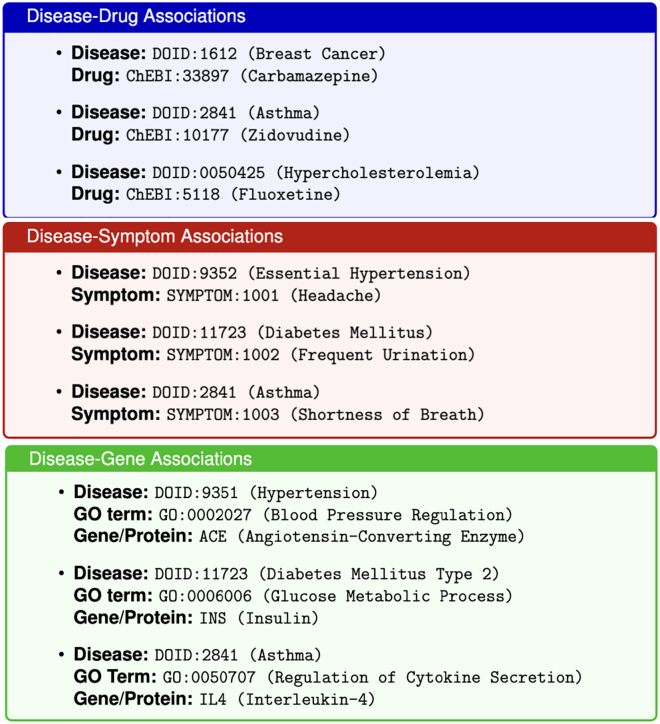
Shows example records from the generated dataset showing the three types of disease-centric associations used in this study The top panel presents disease–drug association pairs. The middle panel displays disease–gene associations. The bottom panel includes disease–symptom associations. The Output was represented in key/value format for further processing and verification.

For the purpose of smooth processing, the prompt also instructed ChatGPT to format the output in JSON format, which was then validated and saved to a file. Figure 5 shows samples containing a few records from each of the three types of associations generated. Specifically, the top panel shows three diseases (breast cancer, asthma, and hypercholesterolemia) and the associated three drugs (Carbamazepine, Zidovudine, and Fluoxetine); the middle panel shows three genes (ACE, INS, and IL4); and bottom panel shows three disease symptoms (headache, frequent urination, and shortness of breath), respectively.

Algorithm 1 shows a one-shot prompt engineering for the task of generating disease-symptom associations.

Algorithm 1 **One-shot prompt engineering for generating disease-symptom associations record in JSON format**

**Require:**: Model M, shot s_1, Number of associations N

 s_1 ← {

  "DOID:11734": "Epistaxis",

  "SYMPTOM:1080": "Nosebleed"}

**Ensure:** Valid and structured JSON output R containing N DOID-SYMPTOM associations.

1: **DEFINE** prompt P:

2:   P ← “You are an assistant that generates 10 DOID-SYMPTOM term associations in a structured JSON format. Ensure the JSON is valid and correctly formatted for parsing. Provide one example in the following format:”

3:   P ← P + s_1

4: **INITIALIZE** response request to model M:

5:  R ← M.generateResponse(P, model = "gpt-4o")

6: PROCESSresponse R:

7: **if** R is a valid JSON format **then**

8:  **Output**R

9: **else**

10:  **Report error**: “Invalid JSON format in response.”

11: **end if**

12: **return** R

#### Term correctness verification against biomedical ontologies

In this work, we address four types of terms that make up the three types of associations that can be represented formally as binary relations.

1. **Disease-drug association:** This type of association is represented as $R_{DD}\subseteq D\times Dr$, where Dr denotes the set of drugs. It signifies the relationship between diseases and the drugs used for their treatment or management.
2. **Disease-symptom association:** This association is denoted as $R_{DS}\subseteq D\times S$, where *S* represents the set of symptoms. It captures the relationship between diseases and their corresponding symptoms.
3. **Gene-process association:** This is expressed as $R_{GP}\subseteq G\times P$, where *P* represents the set of genetic processes. It reflects the relationship between specific genes and the biological processes they are involved in.

**Figure 6:**
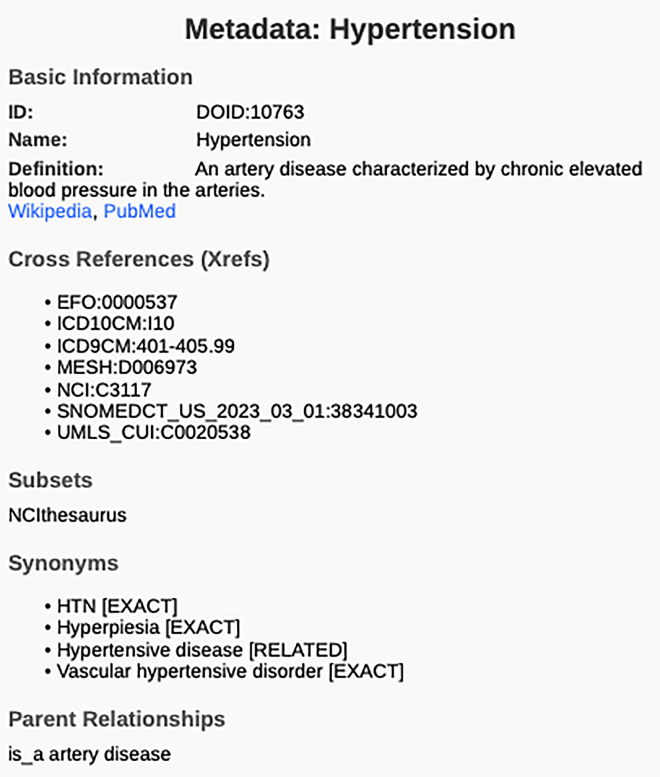
The term “Hypertension,” identified as DOID:10763 in the DOID ontology, includes a metadata item for synonyms The term verification algorithm (Algorithm 2) utilizes this synonyms field to semantically verify the legitimacy of terms generated by ChatGPT.

The terms generated by the model are verified against a specialized ontology. For the diseases, the terms are verified against the the Human Disease Ontology (DOID), the drugs are verified using the Chemical Entities of Biological Interest (ChEBI) ontology, the genetic knowledge is verified using the Gene Ontology (GO), whereas the symptoms are verified using the The Symptom Ontology (SYMP). Since the various ontologies maintain term names and synonyms, the term verification is semantic in nature. Table 5 describes three datasets of associations among disease-symptom, disease-gene, and disease-drug, respectively. Figure 6 shows a proof of a synonym metadata item that describe a DOID:10763 ontology term^47^ known as hypertension and various synonyms namely: (HTN, Hyperpiesia, Hypertensive disease, Vascular hypertensive disorder).

**Table 5:** Datasets generated and used in the experiments

Dataset	Total #
ChatGPT DOID-SYMP JSON Pairs	5466
ChatGPT DOID-GO JSON Pairs	5008
ChatGPT DOID-CHEBI JSON Pairs	2625

It shows the total number of associations for each of three ontology pairings DOID-SYMP, DOID-GO, and DOID-CHEBI. These datasets were validated against trusted ontologies to ensure precision and accuracy. They are designed to support various bioinformatics applications, including disease annotation, genetic process mapping, and drug-disease linkage studies.

Algorithm 2 shows the steps for term verification from the domain ontology. Each ontology term may have a list of synonyms whenever applicable. The algorithm needs to search both the list of terms and the list of synonyms in the ontology to ensure sound and fair verification.

Algorithm 2 **Term verification Using domain-specific ontology**

**Require:** Term instance *t*, Term type *τ*, Domain ontology O

**Ensure:** VERIFIED or UNVERIFIED

1: Let $L_{t}$ be the list of terms in the ontology O

2: **for all** terms $t_{i}\in L_{t}$ **do**

3:  **if** $t=t_{i}$ **then**

4:   **return** VERIFIED

5:  **else**

6:   Let $L_{s}$ be the list of synonyms for *t*

7:   **for all** synonyms $s_{j}\in L_{s}$ **do**

8:    **if** $t=s_{j}$ **then**

9:     **return** VERIFIED

10:    **end if**

11:   **end for**

12:  **end if**

13:  **return** UNVERIFIED

14: **end for**

#### Association reliability verification against biomedical literature

Since the specialized ontologies are term-based resources, they can only support the verification of the terms that make up the associations. Therefore, verification of associations goes beyond the ontology. It is a common practice for scientists to verify biomedical knowledge from the scientific literature. This requires searching digital repositories such as PubMed^54^ and manually verifying whether an evidence holds. The rational behind using PubMed for verification is that PubMed has been recently improved to respond to certain information needs, specifically related to evidence-based medicine and association discovery.^55^

The manual process, though may produce highly accurate results, is labor-intensive and slow in nature. With the large amount of content generated by ChatGPT and other GenAI tools, this process is infeasible. Here, we present an algorithmic approach that offers an automatic means to verify the generated associations. The algorithm is designed to search contextual dataset of biomedical abstracts for a certain association type. To gather this dataset, we performed a contextual search for the three associations: (1) disease and drug, (2) disease and gene, and (3) disease and symptom. Each dataset was used as the basis to verify the corresponding association.

The steps of association verification are outlined in Algorithm 3. The notion of association verification here can be defined as a co-occurrence of the association terms in one of more PubMed abstracts. The number of PubMed abstracts that contain an association is called “coverage”. The number of associations supported by coverage offers a score for a given type of association using the underlying dataset.

Algorithm 3 **Association verification using literature**

**Require:** List of association term pairs $L_{p}$; Dataset of literature *D*

**Ensure:** List of verified associations and their hit ratios, $L_{v}$

1: Initialize $L_{v}\leftarrow Ø$

2: **for all** association term pairs $P\equiv \left(p_{i},p_{j}\right)\in L_{p}$ **do**

3:  Initialize counter←0

4:  **for all** abstracts B∈D **do**

5:   **if** $p_{i}\in B$ **and** $p_{j}\in B$ **then**

6:    counter←counter+1

7:   **end if**

8: **end for**

9: compute hit_ratio $h_{P}\leftarrow \frac{\text{counter}}{\left|L_{P}\right|}$

10: **if** $h_{P}>0$ **then**

11:   append $\left(P,h_{P}\right)$ to $L_{v}$

12:  **end if**

13: **end for**

14: **return** $L_{v}$

### Limitations of the study

This analysis was conducted using data generated by the following ChatGPT models: GPT-4turbo, GPT-4, GPT-4o, and GPT-4o-mini. The associated costs limited the scope of data generation for assessing model consistency, as part of it exceeded the available funding. Future studies should explore the use of open-source LLMs to avoid this limitation.

## Resource availability

### Lead contact

Further information and requests for resources, data, or materials should be directed to and will be fulfilled by the Lead Contact, Ahmed Abdeen Hamed (a.hamed@northeastern.edu).

### Materials availability

All materials generated in this study are available for reproducibility purposes at Zendo DOI [“https://doi.org/10.5281/zenodo.15199189”].

### Data and code availability

• All original code has been deposited at Zenodo under the https://doi.org/10.5281/zenodo.15199189 and is publicly available as of the date of publication.^56^
• Any additional information required to reanalyze the data reported in this article is available from the lead contact upon request.

## Acknowledgments

The work presented in this article has been partly supported by the IBM Faculty Award by the IBM Corporation. Any opinions, findings, and conclusions or recommendations expressed in this article are those of the authors and do not necessarily reflect the views of the IBM Corporation. The authors thank Professor Luis Rocha and the members of the CACSI Laboratory of Binghamton University for the valuable discussion. The authors would like to thank Northeastern University Maimi MSIS students Han Shao, Mengxia Qiu, and Srivarini Mandali for the valuable discussions.

## Author contributions

Conceptualization, A.A.H.; methodology, A.A.H. and B.S.L.; investigation, A.A.H. and A.C.; data curation, M.M.M.; visualization, M.M.M.; writing – original draft, A.A.H.; writing – review and editing, A.A.H., A.C., M.M.M., and B.S.L.; funding acquisition, B.S.L.; supervision, B.S.L.; validation, M.M.M.

## Declaration of interests

The authors declare no competing interests.

## Declaration of generative AI and AI-assisted technologies in the writing process

During the preparation of this work the author(s) used ChatGPT in order to generate associations and simulated articles to produce the datasets of this work. The authors also used ChatGPT to perform LaTeX formatting to enhance the presentation of the work. However, the writing was written entirely by the authors and no GenAI tool was involved in the writing process.

## STAR★Methods

### Key resources table

**Table:** 

REAGENT or RESOURCE	SOURCE	IDENTIFIER
**Software and algorithms**		

OpenAI ChatGPT Model	GPT-4 APIs	https://platform.openai.com/docs/api-reference/models/list
OpenAI ChatGPT Model	GPT-4turbo APIs	https://platform.openai.com/docs/api-reference/models/list
OpenAI ChatGPT Model	GPT-4o APIs	https://platform.openai.com/docs/api-reference/models/list
OpenAI ChatGPT Model	GPT-4o-mini APIs	https://platform.openai.com/docs/api-reference/models/list

**Software and algorithms**		

Source code for the algorithms data of the study	Available on Github and Zenodo	https://doi.org/10.5281/zenodo.15199189

### Method details

All associations were generated using prompt-engineered queries submitted to ChatGPT (GPT-4, GPT-4o, GPT-4o-mini, GPT-4turbo) via the OpenAI API. Each prompt generated disease-centric associations with drugs, genes, or symptoms and returned results in structured JSON format. Results were parsed, stored, and validated against ontologies (DOID, ChEBI, GO, SYMP) using custom Python scripts.

### Quantification and statistical analysis

For each type of association, verification accuracy was calculated by comparing generated terms with corresponding entries in the ontology (literal and synonym match). Verification against PubMed abstracts was performed using co-occurrence statistics. Coverage percentages were reported by period (2009–2014, 2015–2019, 2020–2024), and visualizations were created using matplotlib in Python and model performance comparison was performed and visualized using the JMP (statistical software).^57^

### Additional resources

Prompt templates and analysis scripts used in this study are available at: https://doi.org/10.5281/zenodo.15199189.
